# Retraction Note: Domestic violence and outcome of pregnancy among pregnant females at Alzahraa University Hospital

**DOI:** 10.1038/s41598-025-31011-3

**Published:** 2025-12-02

**Authors:** Abeer A. Almowafy, Mona Alsayed Elkafrawy, Doaa Sadek Ahmed, Samar Samy Ismail, Mahmoud Abdelwahed Alboghdady, Nawal Hamdy Ahmed Keshta

**Affiliations:** 1 International Islamic Institute for Population Studies and Research, Al-Azhar University, Cairo, Egypt; 2 Department of Obstetrics and Gynecology, Faculty of Medicine for Girls, Al-Azhar University, Cairo, Egypt; 3 Community and Occupational Medicine Department, Faculty of Medicine for Girls, Al-Azhar University, Cairo, Egypt; 4 Obstetrics and Gynecology, International Islamic Institute for Population Studies and Research, Al-Azhar University, Cairo, Egypt

Retraction of: *Scientific Reports* 10.1038/s41598-025-16884-8, published online 29 August 2025

The Editors have retracted this Article.

After publication, concerns about some of the results reported in the Article were brought to the attention of Editors, who requested the raw data underlying the study. The Authors provided a transcribed version of the dataset. Post-publication peer review of the file did not resolve the concerns and raised additional issues with respect to the errors in the dataset and how these occurred, as well as proposed corrections and their impact on the published article. It also suggested that the way some of the variables were captured was not realistic, since the study assumed only one possible maternal complication per participant. The Editors therefore no longer have confidence in the results and conclusions presented.

None of the Authors replied to the Editors’ correspondence about this retraction.

